# GAIP Interacting Protein C-Terminus Regulates Autophagy and Exosome Biogenesis of Pancreatic Cancer through Metabolic Pathways

**DOI:** 10.1371/journal.pone.0114409

**Published:** 2014-12-03

**Authors:** Santanu Bhattacharya, Krishnendu Pal, Anil K. Sharma, Shamit K. Dutta, Julie S. Lau, Irene K. Yan, Enfeng Wang, Ahmed Elkhanany, Khalid M. Alkharfy, Arunik Sanyal, Tushar C. Patel, Suresh T. Chari, Mark R. Spaller, Debabrata Mukhopadhyay

**Affiliations:** 1 Department Biochemistry and Molecular Biology, Mayo Clinic, Rochester, Minnesota, United States of America; 2 Department of Pharmacy, King Saud University, Riyadh, Saudi Arabia; 3 Departments of Transplantation and Cancer Biology, Mayo Clinic, Jacksonville, Florida, United States of America; 4 Department of Internal Medicine, Mayo Clinic, Rochester, Minnesota, United States of America; 5 Department of Pharmacology and Toxicology, Geisel School of Medicine at Dartmouth, and Norris Cotton Cancer Center, Lebanon, New Hampshire, United States of America; Medical College of Wisconsin, United States of America

## Abstract

GAIP interacting protein C terminus (GIPC) is known to play an important role in a variety of physiological and disease states. In the present study, we have identified a novel role for GIPC as a master regulator of autophagy and the exocytotic pathways in cancer. We show that depletion of GIPC-induced autophagy in pancreatic cancer cells, as evident from the upregulation of the autophagy marker LC3II. We further report that GIPC regulates cellular trafficking pathways by modulating the secretion, biogenesis, and molecular composition of exosomes. We also identified the involvement of GIPC on metabolic stress pathways regulating autophagy and microvesicular shedding, and observed that GIPC status determines the loading of cellular cargo in the exosome. Furthermore, we have shown the overexpression of the drug resistance gene ABCG2 in exosomes from GIPC-depleted pancreatic cancer cells. We also demonstrated that depletion of GIPC from cancer cells sensitized them to gemcitabine treatment, an avenue that can be explored as a potential therapeutic strategy to overcome drug resistance in cancer.

## Introduction

Macroautophagy, commonly termed as autophagy, is an essential catabolic process that cells implement in diverse biological and physiological activities [1], [2]. Under normal cellular conditions, this process maintains cellular and tissue homeostasis in a protective manner by recycling and degrading cellular components during cell death [2]–[5]. Previously, it was believed that the autophagosome, a double-membraned vesicle, engulfs organelles randomly [1], [2], [5]; however, recent studies have shown that the selection of organelles is directed by cargo specific factors [6]. Additionally, autophagy plays an important role in many disease processes, including cancer [7]. In several cancer types, autophagy can influence the initiation and progression of disease [8], [9] and promote tumor development under the metabolic stress of hypoxia. Because mutations of autophagy-related genes have been reported in human cancers [10], [11], studies have focused on genetic and chemical inhibition of autophagy as a therapeutic strategy [12].

GAIP interacting protein C-terminus (GIPC) was initially identified as an interacting partner of the GTPase-activating protein RGS–GAIP for G-protein coupled receptor subunit GI alpha [13]. The PDZ domain of GIPC stabilizes many transmembrane proteins, including the Glut1 transporter, Semaphorin-F, neuropilin-1, TAX viral protein, dopamine D2 and D3, and IGF1R [14]–[19]. While the majority of PDZ domain-binding ligands for GIPC are transmembrane proteins, several of them are cytosolic proteins, such as APPL1 and RGS19 [13], [20]. Functionally, the N-terminal region of GIPC is involved in dimerization and the C-terminal region of GIPC interacts with myosin VI (MYO6) [21], [22], highlighting its role as an adaptor molecule for loading PDZ domain-targeted cargoes onto the MYO6 motor protein for transport. GIPC is also involved in the trafficking of various transmembrane proteins to endocytic vesicles and essential for the trafficking of internalized integrins during cell migration, angiogenesis, and cytokinesis [23]–[25]. Elevated levels of GIPC expression are reported in several cancers, including pancreatic and breast cancer, promoting their cellular proliferation and survival [19], [26]-[30]. Conversely, depletion of GIPC in cancer cells inhibits proliferation and promotes apoptosis. Knockdown of GIPC results in G_2_ cell-cycle arrest and decreases mortality in MDA-MB231 cells, further suggesting the role of GIPC in cytokinesis and cell migration [23], [31].

Exosomes are intracellular vesicles (40–100 nm) required for intercellular communication in multicellular organisms [23]. The molecular machinery involved in exosome biogenesis includes four multiprotein complexes known as the endosomal sorting complex responsible for transport (ESCRT)-0, -I, -II, and -III, and accessory proteins such as Alix and VPS4. The ESCRT-0, -I, and -II complexes recognize and sequester ubiquitinated membrane proteins at the endosomal restricting membrane, whereas the ESCRT-III complex is accountable for membrane budding and the actual removal of intraluminal vesicles (ILVs) [32]. Recently, Alix (also known as PDCD6IP) was functionally linked to exosome biogenesis through its interaction with the TSG101 and CHMP4 proteins [33]–[35]. A recent study suggests that the formation and release of arrestin domain-containing protein 1-mediated microvesicles (ARMMs) at the plasma membrane depends upon the recruitment of TSG101 protein [36].

There is accumulating evidence that GIPC plays an important role in cellular trafficking. In particular, GIPC acts as a scaffold to control receptor-mediated trafficking [20], [22], [37] and after receptor internalization, GIPC transiently associates with a pool of endocytic vesicles close to the plasma membrane [15]. Exosome biogenesis as well as formation of the autophagosome involves endocytotic vesicles. However, there is no clear evidence that these two mechanisms of vesicle formation crosstalk with each other [38]. In this present study, we reveal a unique regulatory role of GIPC on autophagy through metabolic pathways and the modulation of exosome secretion. We also demonstrate that depletion of GIPC from cancer cells sensitizes them to chemotherapeutic drugs such as gemcitabine, an avenue that can be further explored as a potential therapeutic strategy against drug resistance.

## Materials and Methods

### Cell culture & GIPC knockdown cell lines

Pancreatic cancer cell lines AsPC-1 and PANC-1 were purchased from the American Type Culture Collection (ATCC, Rockville, MD). Cell lines were cultured in RPMI 1640 media (for AsPC-1) or high glucose DMEM (for PANC-1) supplemented with 10% fetal bovine serum (FBS), 5% L-glutamine, and 1% penicillin/streptomycin (Invitrogen, Carlsbad, CA). Cells were maintained at 37°C in an atmosphere containing 95% air-5% CO_2_ (v/v). Stable GIPC knockdown cell lines were generated using lentivirus shRNA. The lentivirus particles were prepared using 293T cells co-transfected with the gag-pol expression plasmid pCMVΔ8.91, the VSVG envelope expression plasmid pMD-G, and the vector plasmid pLKO.1 encoding cDNAs for the expression of GIPC/Synectin shRNA (5′-CCGGGCAAATGCAATAATGCCCTCACTCGAGTGAG-GGCAT-TATTGCATTTGCTTTTTG-3′). GIPC/Synectin shRNA in pLKO.1 was purchased from Open Biosystems. Supernatant was collected 48 h post-transfection and frozen at −80°C. PANC-1 or AsPC-1 cells were then infected overnight at 37°C and stable colonies were isolated after puromycin selection (1 µg/ml). To ensure the efficiency of the GIPC/Synectin knockdown, protein lysates were analyzed by immunoblot for GIPC/Synectin. Control cells were transduced with an empty protein vector. Retroviral pBABE-puro mCherry-EGFP-LC3B plasmid from Addgene (Addgene plasmid 22418) was used to prepare retrovirus particles using 293T cells following standard procedure. AsPC-1 or PANC-1 cells were infected with retrovirus particles and stable colonies were isolated after puromycin selection (1 µg/ml). Experiments were performed at 70–80% cell confluency and confirmed in at least three independent experiments.

### RNA interference, transfection

After a 24-hour incubation with antibiotic-free medium, cells were transfected with anti-GIPC small interfering RNA (siRNA) using the DharmaFECT 2 Transfection Reagent (Dharmacon, Lafayette, CO). Seventy-two to 96 h after transfection, GIPC knockdown was confirmed by Western blot analysis. A similar siRNA approach was adopted for anti-Atg7 and anti-Beclin1 knockdown. For glucose starvation experiments, both control siRNA and GIPC siRNA treated AsPC-1 cells were kept in glucose free RPMI supplemented with 10% FBS for final 16 h of the 96 h experiment. For autophagic flux experiments, both control siRNA and GIPC siRNA treated AsPC-1 and PANC-1 cells were treated with indicated concentrations of Pepstatin-A and E-64d for final 24 h of the 96 h experiment.

### Antibodies and immunoblot analysis

Whole cell lysates were prepared in NP-40 lysis buffer supplemented with a protease inhibitor cocktail (Sigma, St. Louis, MO) and Halt phosphatase inhibitor cocktail (Thermo Scientific, Waltham, MA). Supernatant was collected after centrifugation at 13,000 rpm for 10 min at 4°C and separated by SDS-PAGE. Anti-GIPC, anti-PLCγ, and the horseradish peroxidase-conjugated secondary antibodies were purchased from Santa Cruz Biotechnology. Antibodies against ABCG2, mTOR, phospho-mTOR, p70S6K, phospho-p70S6K, Atg7, Beclin1, AMPK-α, and phospho-AMPK-α were purchased from Cell Signaling Technologies. Anti-CHMP4b and anti-TSG101 was purchased from Abcam; anti-β-actin was purchased from Sigma; and the Alix antibody was purchased from Thermo Scientific. Western blots were developed using the SuperSignal West Pico substrate (Thermo Scientific) and immunoprecipitations were performed as previously described [19].

### Immunofluorescence

Cells (2×10^4^) were seeded on a coverslip in antibiotic-free medium for 24 h. Cells were then transfected with GIPC siRNA or scrambled siRNA (Dharmacon) and the medium was changed 48 h post- transfection. After 96 h, cells were washed and fixed with 4% paraformaldehyde. After blocking with 10% goat serum for 15 min, the cells were permeabilized with 0.2% Triton X-100 at room temperature for 5 min. The slides were then stained with primary antibodies against LC3 for 2 h in 1% goat serum. After incubating the slides with secondary antibodies conjugated to AlexaFluor 488 (1∶200; Life Technologies, Grand Island, NY) for 1 h, slides were mounted with Vectashield (Vector Laboratories, Burlingame, CA) containing 4′,6-diamidino-2-phenylindole (DAPI) and confocal microscopy was performed. In another set of experiments, cells expressing mCherry-EGFP-LC3B were seeded in coverslips and transfected with GIPC siRNA or scrambled siRNA. After 96 h, cells were washed and fixed with 4% paraformaldehyde. Slides were mounted with Vectashield containing DAPI as previously described.

### Glucose uptake and Intracellular glucose measurement assay

Stable cells, either transfected with GIPC shRNA or the control vector, were seeded in 6-well plates and cultured for 48 h. Glucose uptake was measured using the Glucose Uptake Cell-Based Assay Kit (Cayman Chemical, Ann Arbor, MI) using a fluorescently labeled deoxyglucose analog. For an intracellular glucose concentration measurement, the Amplex Red Glucose Assay Kit (Life Technologies) was used with a slight modification to the manufacturer's protocol as described previously [39]. Cells were collected by centrifugation and the resulting cell pellet was washed twice in PBS and dispersed in 1X reaction buffer from the kit. Cells were lysed by probe sonication with three cycles of 10 seconds on, 30 seconds off at 20% power while continuously maintained on ice. Fifty µl of reaction solution (10 mM Amplex Red, 10 U/ml HRP, 100 U/ml glucose oxidase, 50 mM sodium phosphate buffer, pH 7.4) was added to 50 µl of cell lysate in a 96-well plate and incubated in the dark at 37°C for 30 min. The fluorescence (excitation: 544, Emission: 590) was then measured using a SpectraMax plate reader and values were expressed as Relative Fluorescence Units (RFU)/mg protein.

### Exosome isolation

Exosomes were isolated from conditioned medium of PANC-1 and AsPC-1 cells by differential centrifugation. Cells were grown to 70–80% confluency and media was replaced with media containing 10% fetal bovine serum deprived of microparticles through centrifugation (60 min at 100,000×*g*). After 72 h of incubation, supernatants were collected and cleared of cellular debris and dead cells with two sequential spins at 4°C, 3,000×*g* for 10 min. Cleared supernatants were then further centrifuged at 4°C, 60,000×*g* for 70 min. The resulting exosome pellets were washed with phosphate-buffered saline (PBS) solution, and then centrifuged again at 4°C, 100,000×*g* for 70 min. The final exosome pellets were re-suspended in PBS or water depending on the experiment.

### Electron microscopy

Freshly prepared exosomes re-suspended in water were further dispersed in Trump's fixative solution, composed of 4% (v) formaldehyde and 1% (v) glutaraldehyde in 0.1 M phosphate buffer at pH 7.2. The exosomes were then washed with 0.1 M phosphate buffer, 1% osmium tetroxide in 0.1 M phosphate buffer, distilled water, 2% (v) uranyl acetate, distilled water, ethanol, and absolute acetone in sequence. Finally, exosomes were placed on a TEM grid for examination using a *Philips Technai* T12.

### Proteomics analysis

Protein identification was performed via in-gel trypsin digestion using nanoLC-MS/MS with hybrid orbitrap/linear ion trap mass spectrometry. Briefly, protein from the exosomes of GIPC-deficient stable cell lines was resolved on a 4–12% NuPage gel (MOPS buffer) with 20 µl of SDS-PAGE sample buffer containing 50 mM DTT. The gels were stained with BioSafe colloidal blue dye (BioRad) and the desired bands were excised from the gel for mass spectrometry analysis using the following procedures. Colloidal blue stained gel bands were destained in 50% acetonitrile/50 mM Tris pH 8.1 until clear. The bands were then reduced with 50 mM TCEP/50 mM Tris, pH 8.1 at 55°C for 40 min and alkylated with 20 mM iodoacetamide/50 mM Tris pH 8.1 at room temperature for 60 min in the dark. Proteins were digested *in situ* with 30 µl (0.005 µg/µl) trypsin (Promega Corporation, Madison WI) in 20 mM Tris pH 8.1/0.0002% Zwittergent 3–16, at 55°C for 2 h, followed by peptide extraction with 10 µl of 2% trifluoroacetic acid and then 60 µl of acetonitrile. The pooled extracts were concentrated to less than 5 µl on a Speed-Vac concentrator (Savant Instruments, Holbrook, NY) and then brought up in 0.2% trifluoroacetic acid for protein identification by nano-flow liquid chromatography electrospray tandem mass spectrometry (nanoLC-ESI-MS/MS) using a ThermoFinnigan Orbitrap Elite Hybrid Mass Spectrometer (Thermo Fisher Scientific, Bremen, Germany) coupled to an Eksigent nanoLC-2D HPLC system (Eksigent, Dublin, CA). The digested peptide mixture was loaded onto a 250 nl OPTI-PAK trap (Optimize Technologies, Oregon City, OR), custom-packed with Michrom Magic C8 solid phase (Michrom Bioresources, Auburn, CA). Chromatography was performed using 0.2% formic acid in both the A solvent (98% water/2% acetonitrile) and B solvent (80% acetonitrile/10% isopropanol/10% water), and a 2% B to 45% B gradient over 70 min at 300 nl/min through a hand-packed PicoFrit (New Objective, Woburn, MA) 75 µm×200 mm column (Michrom Magic C18, 3 µm). The Orbitrap Elite mass spectrometer experiment was set to perform a FT full scan from 340-1500 m/z with resolution set at 120,000 (at 400 m/z), followed by linear ion trap CID MS/MS scans on the top fifteen ions. Dynamic exclusion was set to 1 and selected ions were placed on an exclusion list for 30 seconds.

### Database searching

Tandem mass spectra were extracted by msconvert (version 3.0.4019; ProteoWizard) and all MS/MS samples were analyzed using Mascot (Matrix Science, London, UK; version 2.4.0), Sequest (Thermo Fisher Scientific; version 27, rev. 12) and X1 Tandem (The GPM, thegpm.org; version CYCLONE (2010.12.01.1)). Mascot, Sequest, and X1 Tandem were set up to search the February 2012 Swissprot database, restricted to human with a decoy reverse database, and assuming the digestion enzyme trypsin. Mascot and X1 Tandem were searched with a fragment ion mass tolerance of 0.60 Da and a parent ion tolerance of 10.0 PPM. Sequest was searched with a fragment ion mass tolerance of 0.60 and a parent ion tolerance of 0.01 Da. Oxidation of methionine and iodoacetamide derivative of cysteine were specified in Mascot, Sequest, and X1 Tandem as variable modifications.

### RNA isolation and Quantitative PCR analysis

Total RNA was isolated from cell lines and exosomes using the miRCURY RNA Isolation Kit – Cell & Plant (Exiqon, Woburn, MA) followed with spectrophotometry (NanoDrop, Thermo Scientific) for quantification and qualitative analysis. Equal amounts of total RNA was reverse-transcribed by oligo (dT) priming using the iScript cDNA Synthesis kit (Bio-Rad, Hercules, CA) following the manufacturer's instructions. Real-time PCR was performed using the ABI 7500 Real-Time PCR System (Applied Biosystems, Foster City, CA) and the SYBR Green PCR Master Mix (Applied Biosystems) as described previously [40]. Glut1 and β-Actin primers were purchased from SABiosciences (Frederick, MD).

### Drug sensitivity assay

Briefly, 5×10^3^ cells were seeded per well in triplicate, in 96-well flat-bottom plates with 100 µl of medium. After 24 h, variable concentrations of gemcitabine (µg/ml) were added and the cells were incubated for an additional 72 h. At the end of the treatment period, 20 µl of MTS solution containing PMS (MTS: PMS  = 20∶1 vol. ratio) were added to each well and the cells were incubated at 37°C for 1 to 2 h. The absorbance at 490 nm was recorded using a SpectraFluor PLUS (Molecular Devices, Sunnyvale, CA) and the half maximal inhibitory concentration (IC50) values were calculated as concentrations corresponding to a 50% reduction of cellular growth. Prior to the drug sensitivity testing, cell viability was determined by the MTS assay (Promega, Madison, WI).

### Statistical analysis

The data in the bar graphs represent the mean ± standard deviation of at least three independent experiments, each performed with triplicate samples. Statistical analyses were performed using a Student's t test, with a two-tailed value of P<0.05 to be considered significant.

## Results

### GIPC depletion induces autophagy in pancreatic cancer cells

**Figure 1:**
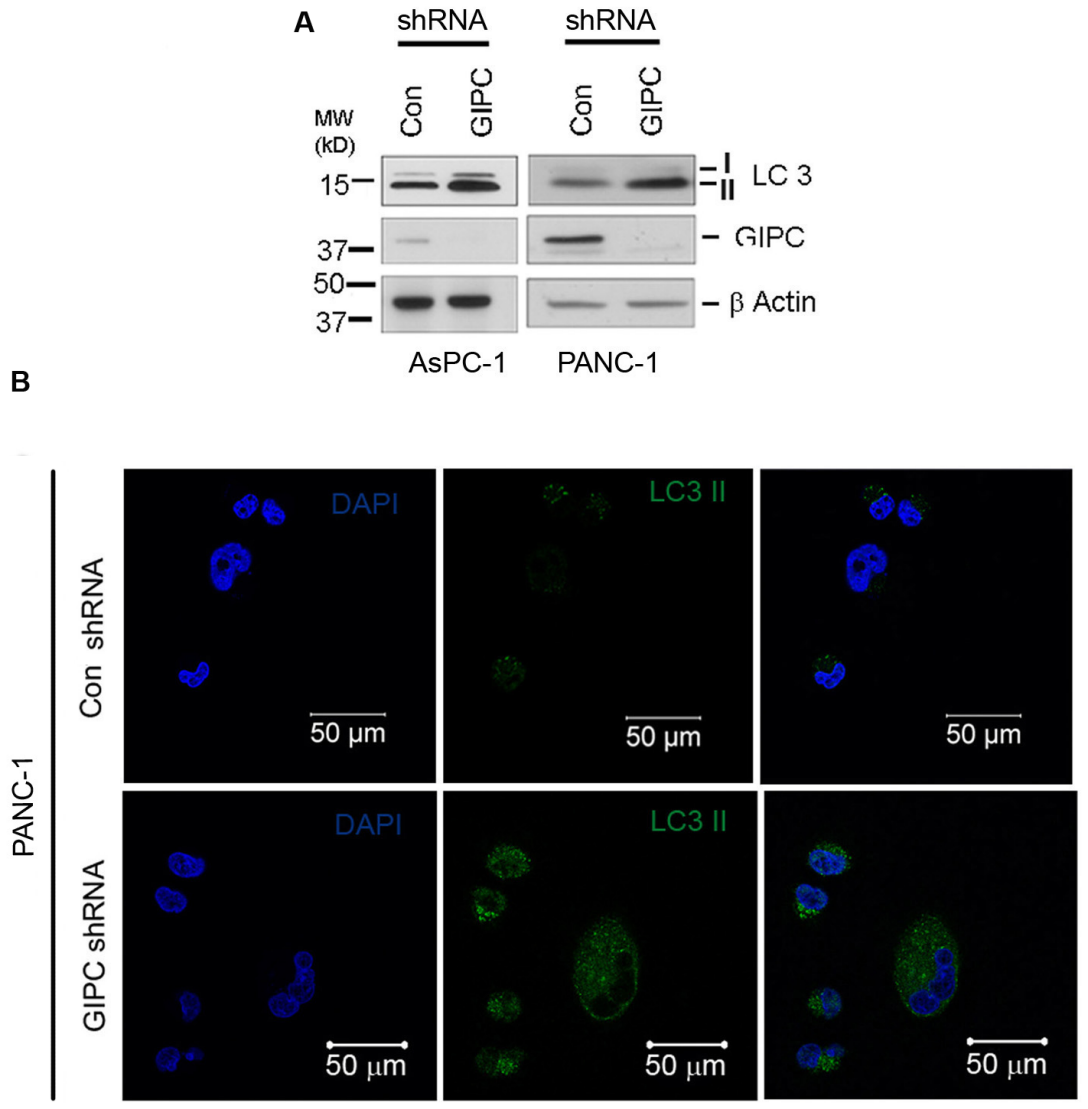
GIPC induce autophagy in the pancreatic cancer cells. A) AsPC-1 and PANC-1 cells were infected with lentiviruses expressing shRNAs to GIPC and scrambled control. An equal amount of whole-cell lysates from AsPC-1 and PANC-1 GIPC depleted cells were analyzed by immunoblotting (IB) with the antibodies for GIPC and LC3II. β-Actin is used as loading control. B) A representative immunofluorescence analysis of PANC-1 cells for expression of LC3 II (green) in GIPC depleted PANC-1 cells compared to the control cells. Cells were counterstained with DAPI (blue).

Utilizing the GIPC-depleted AsPC-1 and PANC-1 pancreatic cell lines, we investigated whether GIPC modulated autophagy by assessing the autophagy-related microtubule-associated protein light chain 3 (LC3) conversions (LC3-I to LC3-II) via Western blot analysis. It is well known that the conversion of the light chain 3-I (LC3-I), upon conjugation to phosphatidylethanolamine (PE), forms the conjugate light chain 3-II (LC3-II) which is then recruited to the membranes of autophagosomes [13], [20]. LC3 expression has been widely used to monitor and establish the status of autophagy as the amount of LC3II correlates with the number of autophagosomes [41]. After a thorough investigation of the LC3-II level in the pancreatic stable cell lines, we observed an elevated LC3-II level in cells deficient for GIPC, indicating the activation of autophagy (Figure 1A). We also observed an increase in LC3-II (green) puncta formation in the GIPC-depleted cells by immunofluorescence study (Figure 1B). GIPC knockdown in presence of lysosomal protease inhibitors, Pepstatin-A and E-64d, further increased LC3-II levels in a dose-dependent manner compared with GIPC knockdown alone, indicating enhancement of autophagic flux (Figure S1A). Furthermore, we used a tandem fusion protein mCherry-EGFP-LC3B containing acid-insensitive mCherry and acid-sensitive EGFP as an autophagic flux reporter system [42], [43]. During autophagosome formation, both EGFP and mCherry are detected in autophagosomes which appear as yellow puncta. However, once autophagosomes fuse with lysosomes, the green fluorescence is lost because of the degradation of EGFP by acid lysosomal proteases resulting only red puncta. Therefore, presence of both yellow and red puncta indicates a functional autophagic flux process. Here we have used both AsPC-1 and PANC-1 cell lines stably expressing mCherry-EGFP-LC3B to show the increase in both yellow and red puncta upon GIPC knockdown which also indicated an increase in autophagic flux (Figure S1B). These findings suggested that GIPC knockdown induces the formation of autophagosomes in pancreatic cancer cells.

**Figure 2:**
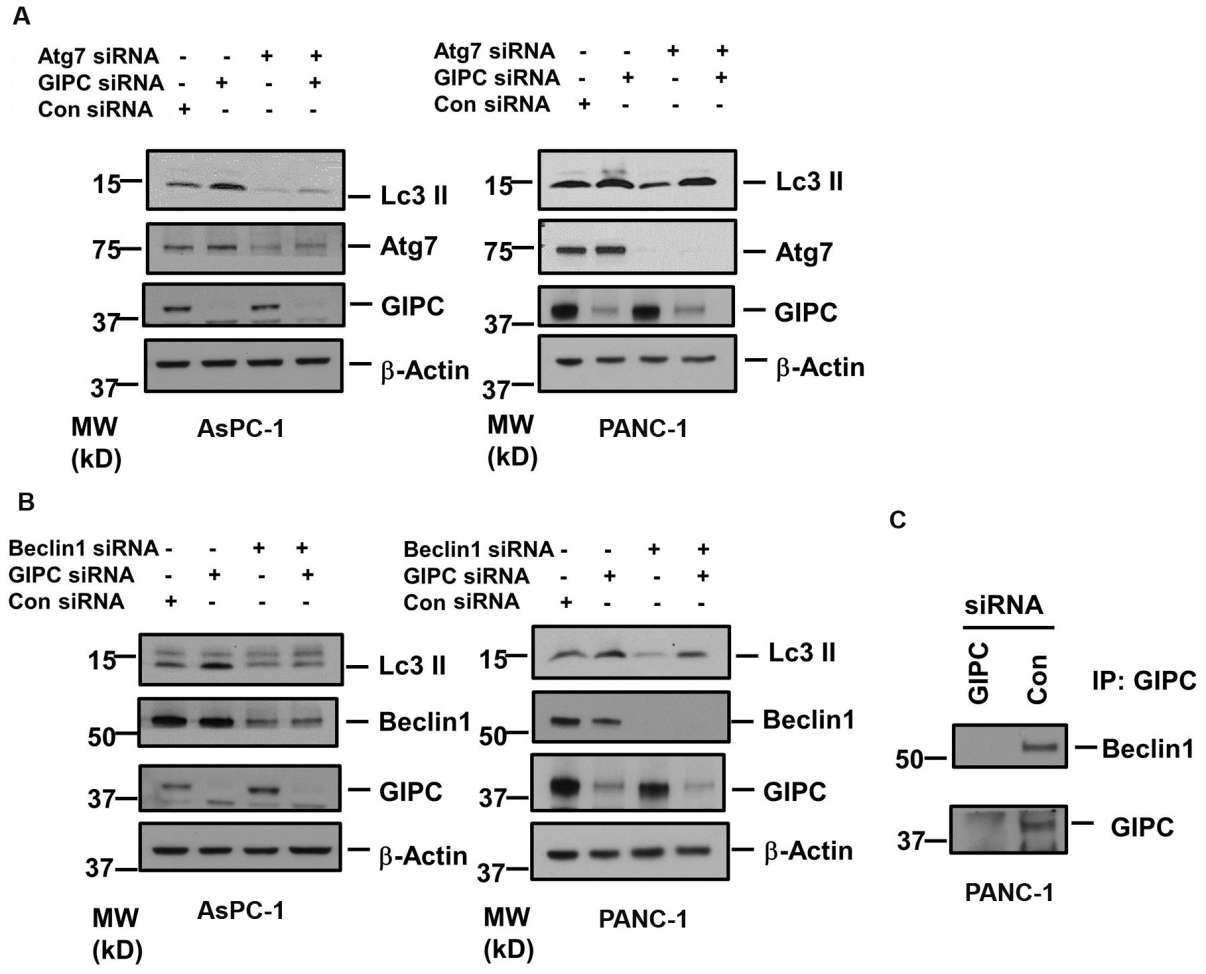
The role of Beclin 1 and Atg 7 in GIPC induced autophagy. A) Immunoblot analysis of the Atg7 expression in AsPC-1 and PANC-1 cell lysates transfected with siRNA to GIPC, Atg7 and scrambled control. β-Actin is used as loading control. B) Immunoblot analysis of the Beclin1 expression in AsPC-1 and PANC-1 cell lysates transfected with siRNA to GIPC, Beclin1, and scrambled control. β-Actin is used as loading control. C) Co-immunoprecipitation (IP) of the PANC-1 cell lysates transfected with siRNA to GIPC, and scrambled control using GIPC antibody. Immunocomplexes were analyzed by immunoblotting (IB) with antibodies to Beclin1 and GIPC.

We further investigated the effect of two autophagy-related genes, Atg7 and Beclin1, on GIPC-mediated autophagic regulation. To assess the interaction of Atg7 and Beclin1, we reduced the level of Atg7 and Beclin1 by RNA interference (RNAi) in both PANC-1 and AsPC-1 cells. As shown in Figures 2A and 2B, we did not observe any significant change in Atg7 or Beclin1 expression after GIPC depletion in both pancreatic cancer cells. As Atg7 and Beclin1 are two key components for autophagosome biogenesis, we also observed a decrease in LC3 II conversion from LC3 I upon knockdown of Atg7 and Beclin1 in both the pancreatic cancer cells. In AsPC-1 cells, we noticed that induction of autophagy with depletion of GIPC was significantly impeded by reduction of Atg7 and Beclin1. On the contrary, in PANC-1 cells, Atg7 and Beclin1 could not affect the LC3II conversion subject to GIPC depletion. We further explored the association of GIPC with Atg7 and Beclin1 by co-immunoprecipitation experiments and found Beclin1 to be in the same complex with GIPC (Figures 2C) but did not get conclusive result for Atg7 (data not shown).

### GIPC mediates autophagy through metabolic stress pathways

**Figure 3:**
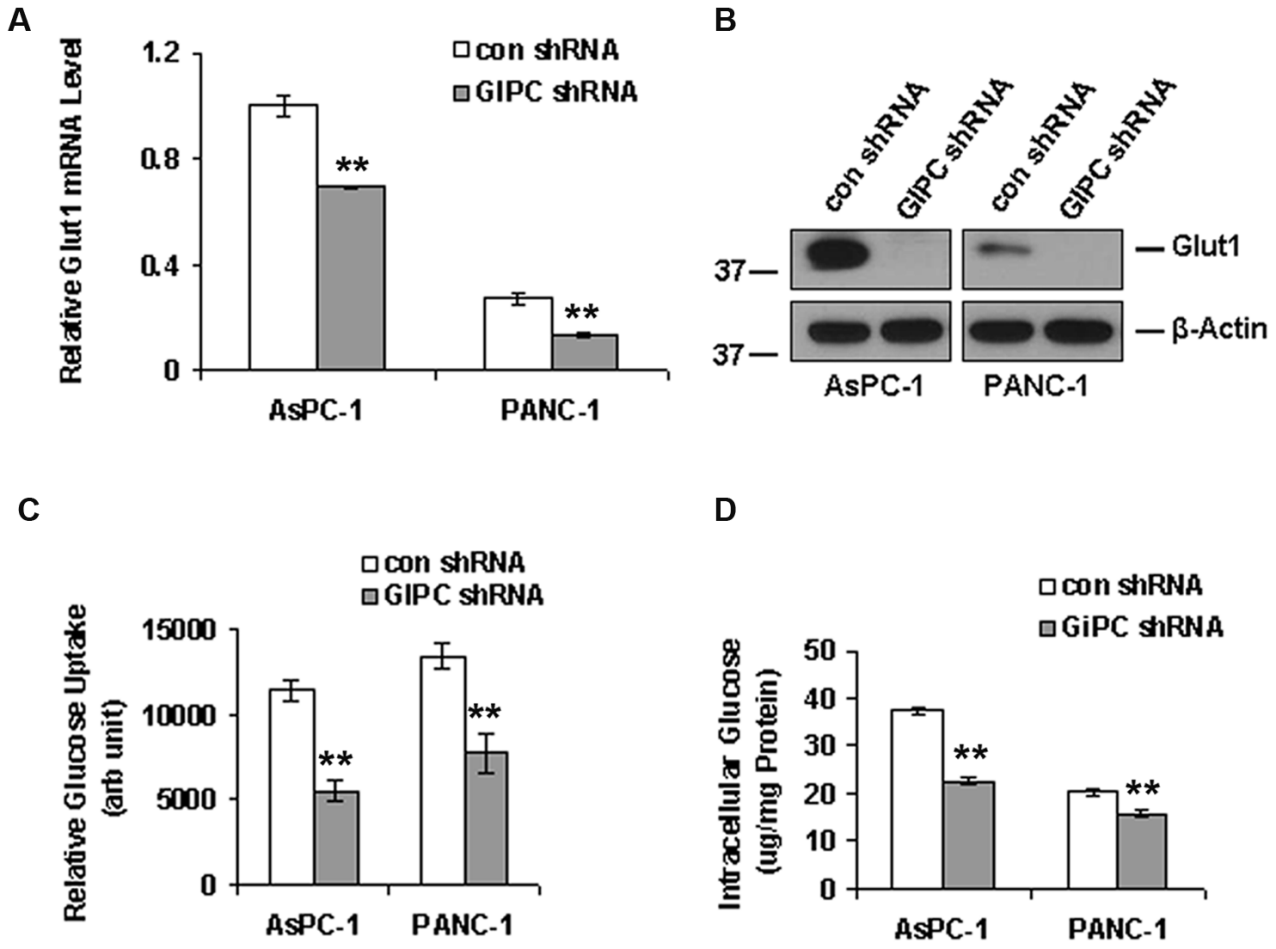
GIPC regulates autophagy by interfering with glucose uptake. A) Quantitative PCR and B) Western blot analysis of Glut1 to analyze the effect of GIPC-depletion in Glut1 expression. β-Actin is used as loading control. Both Glut1 mRNA and protein levels decreased significantly upon GIPC depletion in AsPC-1 and PANC-1 cells. C) Glucose uptake was significantly decreased in GIPC depleted cells as compared to the control cells in AsPC-1 and PANC-1 cells (** denotes p<0.01). D) Intracellular glucose levels were also significantly decreased in the GIPC depleted AsPC-1 and PANC-1 cell lines confirming the role GIPC in glucose metabolism (** denotes p<0.01).

**Figure 4:**
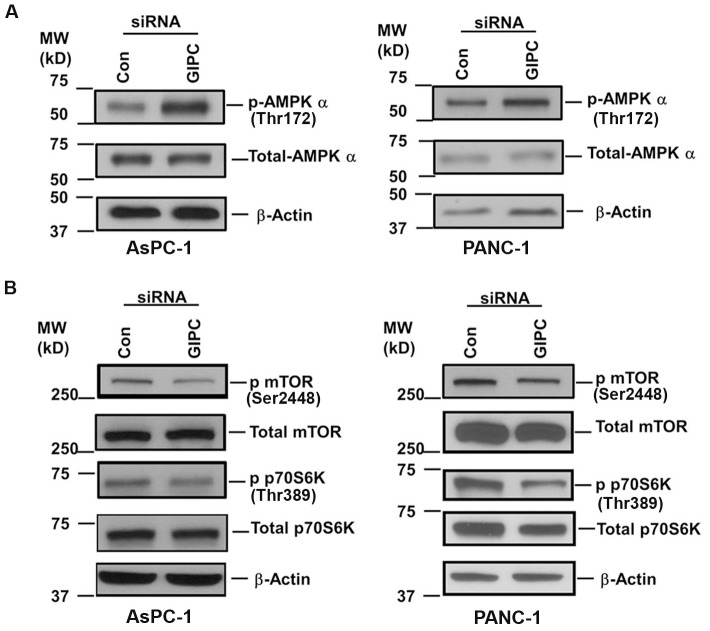
GIPC regulates stress induced metabolic pathways. A) Immunoblot of the cell lysates from GIPC depleted and control AsPC-1 and PANC-1 cells are being probed with p-AMPK-α, total AMPK-α. β-Actin is used as loading control. B) Further, immunoblot of cell lysates from above condition were being probed with p–mTOR, total mTOR, p-p70S6K and total p70S6K. β-Actin is used as loading control.

Glut1 is associated with glucose uptake in cancer cells and GIPC is known to stabilize Glut1 in the cell membrane as a PDZ domain-containing interaction partner [14]. In this regard, we examined whether knocking down GIPC in pancreatic cancer cells would destabilize Glut1 and disrupt glucose uptake into these cells. As expected, we found a significant decrease in Glut1 expression in both mRNA and protein level upon GIPC knockdown in AsPC-1 and PANC-1 cells (Figure 3A & 3B). Furthermore, we found that the relative glucose uptake for AsPC-1 and PANC-1 cells was significantly reduced in the absence of GIPC, compared to that of control cells (Figure 3C). To determine whether intracellular levels of glucose were also dependent upon the status of GIPC, we monitored the intracellular glucose level after GIPC knockdown in the same pancreatic cancer cell lines and found levels to be significantly reduced when compared to wild type cells (Figure 3D). Importantly, under stress conditions, cellular AMP usually regulates the intracellular glucose level. AMP levels were elevated in glucose starvation, which, in turn, further activated the kinase activity of AMPK-α through phosphorylation [44], [45]. To investigate this mechanism in pancreatic cancer cell lines, we examined the AMPK-α status by immunoblot in GIPC stable knockdown cells. Our results revealed a high level of phosphorylated AMPK-α upon GIPC depletion (Figure 4A), suggesting that GIPC may modulate the AMPK pathways.

We further investigated the molecular mechanism of autophagy by examining downstream molecules of the AMPK-α pathway. We observed decreased levels of mTOR phosphorylation after GIPC knockdown in AsPC-1 and PANC-1 cells; however, total mTOR expression did not change. Additionally, we observed a decrease in a known downstream effector of mTOR, the phospho-p70S6K to p70S6k ratio, in GIPC-depleted cell lysates compared to the control parental cells (Figure 4B). Removal of extracellular glucose further enhanced AMPK-α phophorylation and reduced mTOR phosphorylation as well as p70S6K phosphorylation (Figure S2). However, LC3 levels were decreased upon removal of extracellular glucose which corroborates with previous reports [46] suggesting extracellular glucose removal kills the cells either by apoptosis or necrosis in stead of inducing autophagy as a prosurvival effect. Taken together, our results suggest that GIPC controls autophagy through the regulation of metabolic pathways in pancreatic adenocarcinoma cells.

### GIPC influences exosome secretion and biogenesis

**Figure 5:**
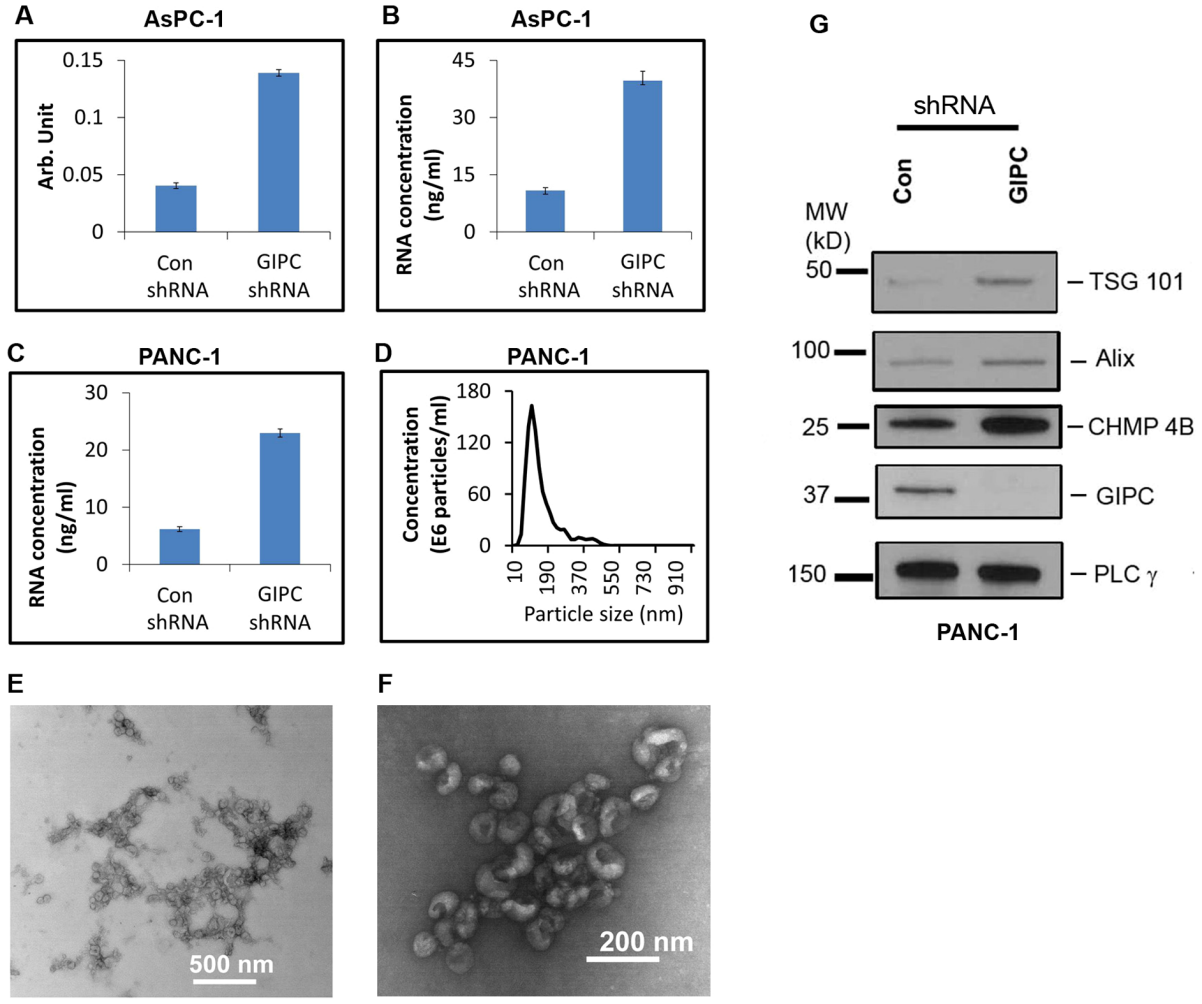
GIPC increase exosome secretion and biogenesis. Exosomes were isolated from culture media of AsPC-1 and PANC-1 GIPC depleted and control cell culture. For qualitative measurement same amount of cells were seeded in culture plates. A) A comparison of activity of acetylcholine esterase is depicted for exosomes collected from GIPC depleted and control AsPC-1 cell line. B&C) A comparison of total RNA content of the exosome preparation from AsPC-1 and PANC-1 cell culture is displayed. D) A representative size distribution profile of the exosome preparation is obtained using Nanosight. E) A representative transmission electron micrograph (TEM) of exosomes is presented in this figure. Scale bar is 500 nm. F) A higher magnification TEM image of exosomes is presented. The scale bar is 200 nm. G) Immunoblot conducted with cells lysates collected from GIPC knockdown cells as well as control cells were being probed with TSG 101, Alix, and CHMP 4B. PLC γ is used as loading control.

With the exosomes collected from the stable transfectants, we performed enzymatic assays for acetylcholine esterase activity as described previously [14]. This assay revealed a greater abundance of exosomes in the conditioned media of GIPC-deficient cell lines. A 3.5 or greater fold increase in exosome production was observed in conditioned media collected from GIPC-depleted AsPC-1 cells compared to the control (Figure 5A). We obtained similar result with GIPC-depleted PANC-1 cells as well (data not shown). We also determined the concentration of total RNA in these exosomes as another measure of exosome abundance and found similar results (Figures 5B & 5C). Nanoparticle tracking analysis using the NanoSight LM10 confirmed the size distribution of our exosome preparations. With a mode of approximately 100 nm, their size was consistent with the current exosome definition (Figure 5D).

We then performed morphological characterization of the exosome preparation with an ultra-structural analysis of the exosome pellets by heavy metal negative staining and transmission electron microscopy (TEM). Analysis of the TEM images confirmed the exosome dimensions in our samples. Figure 5E represents the typical morphology of the overall exosome population in a lower TEM magnification. Further analysis of the TEM images at higher magnification confirmed the typical cupped shape structure of exosomes (Figure 5F). These analyses confirmed that the presence or absence of GIPC did not affect exosome morphology.

To confirm whether the increased exosomes in GIPC-depleted cells correlated with activation of the exosome biosynthesis machinery, we checked the expression of key genes (Alix, TSG101, CHMP4B) involved in exosome biogenesis by immunoblot. We observed an increased expression of Alix, TSG101, and CHMP4B in GIPC knockdown cells when compared to control cells (Figure 5G).

### GIPC influences exosome content and sensitizes pancreatic cancer cell lines to chemotherapeutic drugs

**Figure 6:**
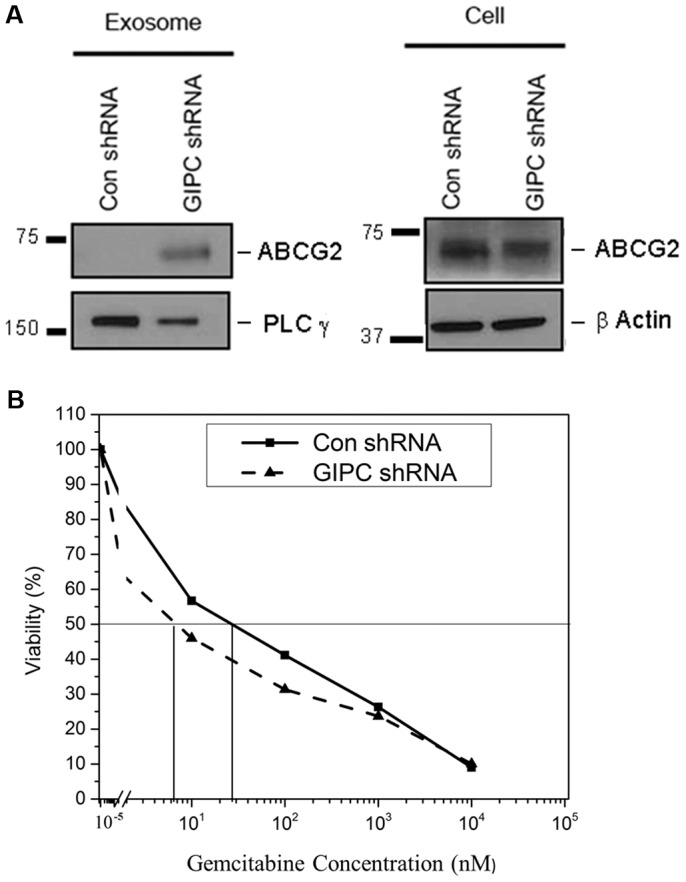
GIPC modulates expression of drug resistance associated gene *ABCG2* and sensitizes pancreatic cancer cell lines to gemcitabine. A) ABCG2 expression was confirmed at protein level by western blot in GIPC knockdown and control cells as well as in corresponding exosomes. PLC γ is used as loading control for exosomes and β-Actin is used as loading control for cell lysates. B) GIPC +/- PANC-1 cells were treated with different concentration of the gemcitabine for 72 h. Effect of the drug treatment was evaluated using MTS cell viability assay. The horizontal bar represents the IC50 level.

To compare exosome content in GIPC knockdown and wild type cells, we performed proteomics analyses on the exosomes collected from the PANC-1 stable cell lines. For proteome analysis, protein was extracted from the secreted exosomes and we found that the content of exosomes greatly varied depending on GIPC status. In support of the robustness and sensitivity of our analysis methods, proteomics data confirmed the absence of GIPC protein in exosomes isolated from the GIPC deficient cells but not in the control samples. This also demonstrated for the first time the presence of GIPC in exosomes. Furthermore, proteomic analysis of the exosomes isolated from the PANC-1 stable cells revealed a significant enrichment of genes involved in drug resistance (data not shown). Among these genes, the most notable was the ATP-binding cassette sub-family G member 2 (ABCG2). Mass spectrometry analysis identified ABCG2 to be overexpressed in GIPC deficient exosomes by 13 fold when compared to control exosomes (data not shown). We have verified this observation for exosomes at the protein level as shown in Figure 6A. We did not observe any change in ABCG2 expression in cell lysates.

To verify the role of ABCG2 in drug sensitivity, we tested gemcitabine, a frontline pancreatic cancer drug, at different concentrations in GIPC-depleted PANC-1 cells. Our results show that gemcitabine treatment sensitized the GIPC deficient PANC-1 cells by decreasing the IC50 values of the drug from 26 nM to 6 nM. The results suggest the involvement of GIPC in pancreatic cancer drug response and make more resistance phenotypes (Figure 6B).

## Discussion

GIPC has already been identified as an important regulatory molecule for stabilizing transmembrane proteins. It acts as a scaffold to control receptor-mediated trafficking [20], [22], [37]. Following receptor internalization, GIPC transiently associates with a pool of endocytic vesicles close to the plasma membrane [15]. Because GIPC is directly involved in the trafficking of endocytotic vesicles, it was therefore logical to investigate its influence on autophagy. In this work, we report a novel role for GIPC as a master regulator for both autophagy and exosome biogenesis. We show in pancreatic cancer cells that depletion of GIPC created an environment of metabolic stress. This, in turn, induced autophagy and microvascular shedding. We also observed that the GIPC status determined the cellular message sent to the extracellular space via exosome secretion.

To perform this study, stable GIPC-deficient pancreatic cancer cell lines were generated and the status of autophagy was monitored by assessing the expression of LC3-II, a protein that serves as a marker for autophagy. The increase in LC3-II expression as well as the abundance of LC3-II positive vesicles in GIPC-deficient cells clearly illustrated the involvement of GIPC in autophagy.

However, an increase in LC3-II level or a greater number of LC3-II positive vesicles cannot confirm whether autophagosome formation is upregulated or autophagic degradation is blocked. Therefore, we performed autophagic flux experiment in presence of lysosomal protease inhibitors [47]. A further increase in LC3-II levels were observed in presence of lysosomal protease inhibitors, indicating that GIPC knockdown was really inducing autophagosome formation. Increased abundance of both yellow and red LC3-II puncta in AsPC-1 and PANC-1 cells expressing mCherry-EGFP-LC3B upon GIPC knockdown also confirmed this observation.

We further investigated the role of the autophagy-associated genes *Beclin1* and *Atg7* in our stable pancreatic cell lines. Our results show that the expression of Beclin1 and Atg7 did not change in GIPC-deficient cells. These findings suggest that GIPC induced autophagy through an alternate mechanism independent of Beclin1 and Atg7.

Rapidly growing and proliferating cells, such as cancer cells, require elevated metabolism. Cancer cells for biosynthesis and energy production, in particular, preferentially consume glucose. Glucose uptake is an essential step in glucose metabolism and is achieved by facilitative glucose transporters (Glut family members). Glut1 facilitates glucose transport across the plasma membranes of mammalian cells [21], [48], [49] and helps maintain the low-level basal glucose uptake required to sustain respiration in all cells. GIPC is known to interact with many transmembrane proteins, including Glut1, through the C-terminal PDZ domain-binding motif and help in their stabilization [14]. With GIPC depletion, Glut1 expression and glucose uptake decreases. We observed a similar phenomenon in our pancreatic cancer cell lines where Glut1 expression as well as glucose uptake and intracellular glucose levels dropped with GIPC knockdown. With this glucose deprivation, we further observed high levels of phosphorylated AMPK-α During nutrient deprivation and metabolic stress, AMPK is allosterically activated by an elevated intracellular AMP/ATP ratio [50], followed by the phosphorylation of threonine 172 within its α subunit [44]. Additionally, AMPK is known to negatively regulate mTOR signaling [45], [51]–[54] and we observed decreased phosphorylation of mTOR after activation of AMPK in the GIPC-deficient cells. Under different stress situations, a linear relationship exists between the degree of phosphorylation of ribosomal protein S6 and the percentage of inhibition of autophagic proteolysis [55]. Our results are in agreement with already published data and we have observed a similar effect with p70S6K. Removal of extracellular glucose further increased AMPK-α phosphorylation and reduced phosphorylation of both mTOR and p70S6K. However, LC3 levels were decreased upon extracellular glucose removal which suggests that not all forms of starvation induce autophagy [46].

Previous studies have reported that GIPC plays an important role in cellular trafficking by acting as a scaffold. There is substantial evidence that after receptor internalization, GIPC transiently associates with a pool of endocytic vesicles close to the plasma membrane. The known role of GIPC in cellular trafficking prompted us to hypothesize that GIPC may play a role in exosome secretion. We prepared exosomes from the stable GIPC deficient cell lines and their wild type controls. To confirm the quality of our exosome preparation, we measured the abundance of exosomes by acetylcholine esterase enzymatic assays and RNA quantification. Despite the conflicting reports in the literature regarding the definition of microvesicles and exosomes, our transmission electron micrograph (TEM) and NanoSight results confirmed that our samples contained exosomes based on size (40 to 100 nm) and morphology.

In this study, we report increased exosome secretion after GIPC knockdown. This observation was not only confined to pancreatic cancer cell lines but also in the renal cancer cell line 786-O (data not shown). Because the same number of cells was plated for exosome collection, the variation in exosome secretion was attributed to the influence of GIPC. We further investigated the status of exosome biogenesis in these cells. Exosome production is initiated by the formation of MVEs and budding through the plasma membrane. In this study, we observed that in the absence of GIPC, the expression of Alix, TSG101, and CHMP4B increased in comparison to the GIPC control cells. These findings suggest that in absence of GIPC, the MVE synthesis machinery is overactive and stimulates exosome secretion.

In addition to exosome secretion and biogenesis, the molecular composition of the exosomes varied significantly in physiological and disease states. Therefore, we investigated the effect of GIPC depletion at the protein level by mass spectrometry analysis. Interestingly, we identified the drug resistance gene ABCG2 to be significantly upregulated in our exosome preparations and evaluated the effect of gemcitabine on GIPC depleted and control cells. We found an increased sensitivity to gemcitabine in GIPC-deficient pancreatic cancer cells.

In summary, GIPC modulates autophagy in pancreatic cancer cells through the metabolic pathways and glucose deprivation. GIPC not only controls exosome biogenesis but also influences exosome content. Most likely, the absence of GIPC promotes the depletion of the drug resistance molecule ABCG2 through exosome exocytosis. As a result, GIPC-deficient cells become more drug sensitive. Alternatively, depletion of GIPC in cancer cells may result in the sequestering of ABCG2 in vesicles, rendering it inaccessible and therefore, nonfunctional. This then sensitizes the cells to gemcitabine. These findings can be further explored as a novel therapeutic approach to overcome the drug resistance so often observed in cancers.

## Supporting Information

Figure S1 **GIPC knockdown induces autophagic flux.** A) GIPC knockdown in presence of lysosomal protease inhibitors showed an increase in LC3-II levels compared with GIPC knockdown alone in both AsPC-1 and PANC-1 cells indicating an increase in autophagic flux. PLCγ is used as loading control. B) AsPC-1 and PANC-1 cell lines stably expressing mCherry-EGFP-LC3B showed an increase in both yellow and red puncta upon GIPC knockdown which indicated an increase in autophagic flux. Scale bar  = 50 µm.
(DOC)

Figure S2 **Glucose Starvation does not induce autophagy.** Removal of extracellular glucose engages starvation signals by increasing AMPK-α phosphorylation and decreasing phosphorylation of both mTOR and p70S6K in AsPC-1 cells. However, LC3 levels were decreased upon removal of extracellular glucose.
(DOC)
