# Supplementary material for: GAIP Interacting Protein C-Terminus Regulates Autophagy and Exosome Biogenesis of Pancreatic Cancer through Metabolic Pathways
Source: PLoS One. 2014 Dec 3;9(12):e114409. doi: 10.1371/journal.pone.0114409 (PMC4255029; doi:10.1371/journal.pone.0114409)
Supplement: Figure S1 — GIPC knockdown induces autophagic flux. A) GIPC knockdown in presence of lysosomal protease inhibitors showed an increase in LC3-II levels compared with GIPC knockdown alone in both AsPC-1 and PANC-1 cells indicating an increase in autophagic flux. PLCγ is used as loading control. B) AsPC-1 and PANC-1 cell lines stably expressing mCherry-EGFP-LC3B showed an increase in both yellow and red puncta upon GIPC knockdown which indicated an increase in autophagic flux. Scale bar = 50 µm. (DOC) [file pone.0114409.s001.doc]

**
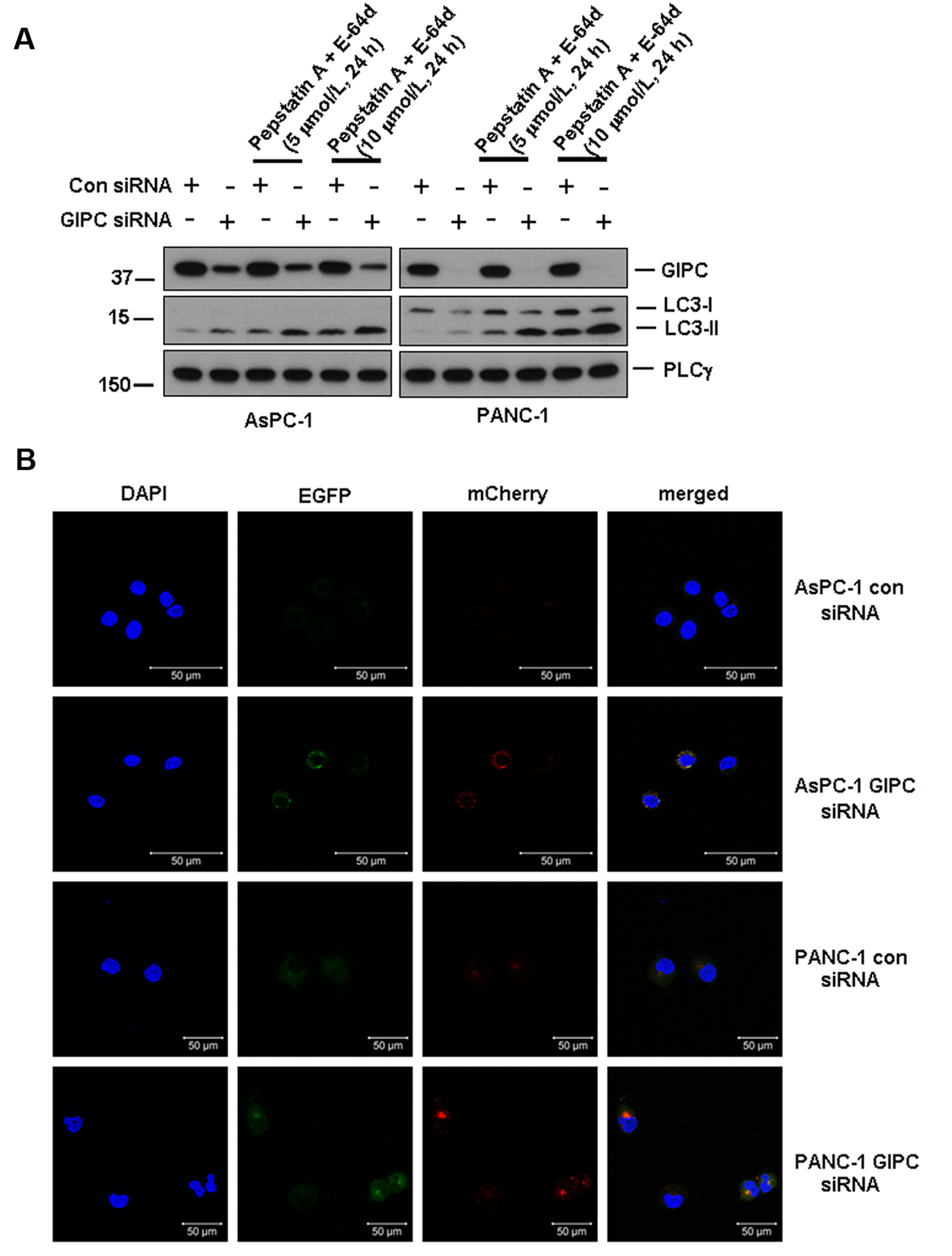
**

**Supplementary Figure S1: GIPC knockdown induces autophagic flux.**

A) GIPC knockdown in presence of lysosomal protease inhibitors showed an increase in LC3-II levels compared with GIPC knockdown alone in both AsPC-1 and PANC-1 cells indicating an increase in autophagic flux. PLCγ is used as loading control. B) AsPC-1 and PANC-1 cell lines stably expressing mCherry-EGFP-LC3B showed an increase in both yellow and red puncta upon GIPC knockdown which indicated an increase in autophagic flux. Scale bar = 50 μm.
