# Supplementary material for: GAIP Interacting Protein C-Terminus Regulates Autophagy and Exosome Biogenesis of Pancreatic Cancer through Metabolic Pathways
Source: PLoS One. 2014 Dec 3;9(12):e114409. doi: 10.1371/journal.pone.0114409 (PMC4255029; doi:10.1371/journal.pone.0114409)
Supplement: Figure S2 — Glucose Starvation does not induce autophagy. Removal of extracellular glucose engages starvation signals by increasing AMPK-α phosphorylation and decreasing phosphorylation of both mTOR and p70S6K in AsPC-1 cells. However, LC3 levels were decreased upon removal of extracellular glucose. (DOC) [file pone.0114409.s002.doc]

**
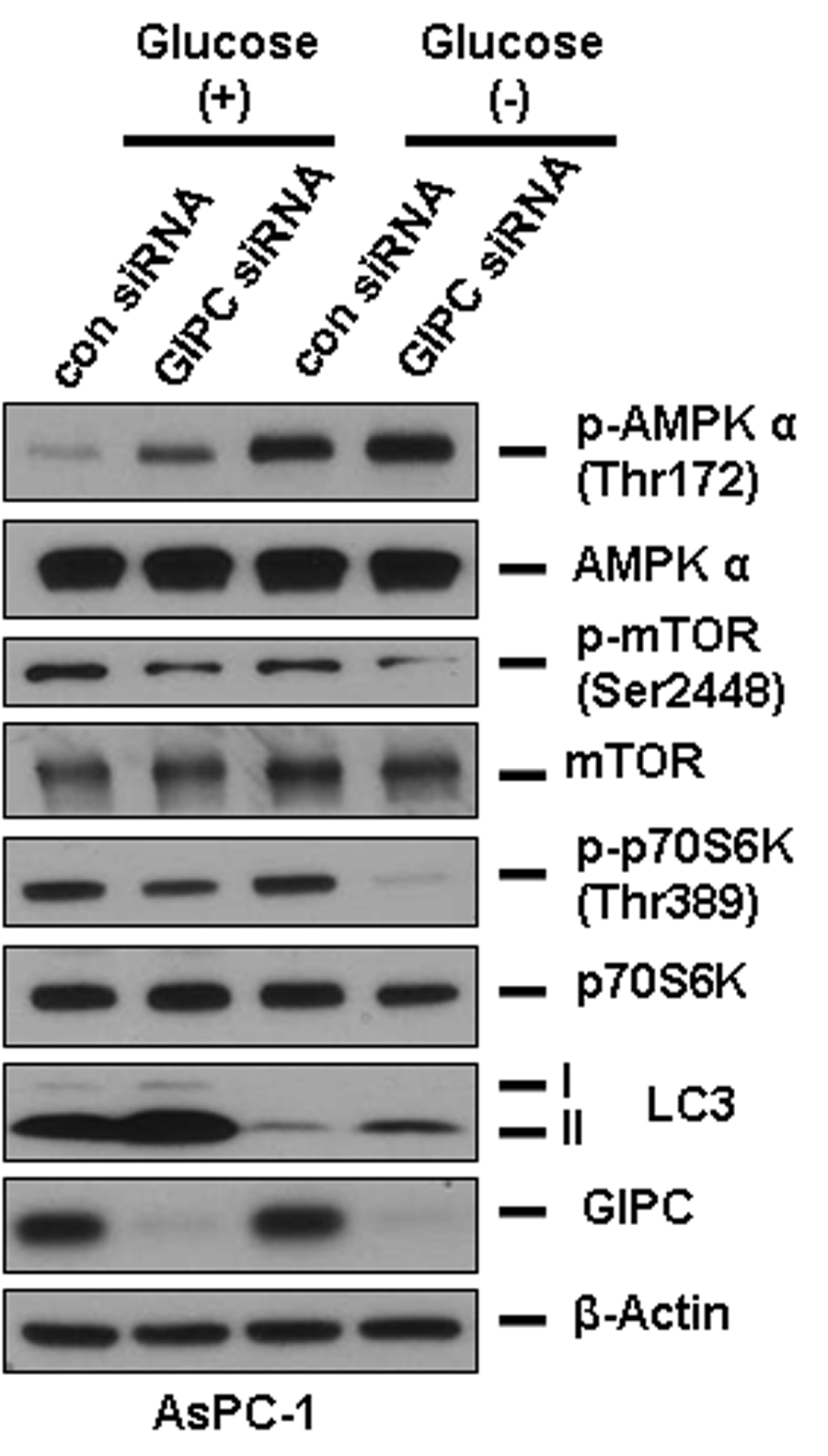
**

**Supplementary Figure S2: Glucose Starvation does not induce autophagy.**

Removal of extracellular glucose engages starvation signals by increasing AMPK-α phosphorylation and decreasing phosphorylation of both mTOR and p70S6K in AsPC-1 cells. However, LC3 levels were decreased upon removal of extracellular glucose.
